# Cytotoxic Potential of Novel Quinoline Derivative: 11-(1,4-Bisaminopropylpiperazinyl)5-methyl-5H-indolo[2,3-b]quinoline against Different Cancer Cell Lines via Activation and Deactivation of the Expression of Some Proteins

**DOI:** 10.3390/ijms241814336

**Published:** 2023-09-20

**Authors:** Sara Fathy Abd Elrahman, Abdullah A. S. Ahmed, Doaa Abd Elsatar, Salma Elkady, Amira Elgendy, Fatma Alnakeeb, Elshaymaa I. Elmongy, Hanan A. Henidi, Saad M. El-Gendy, Ibrahim El Tantawy El Sayed, Ahmed A. El-Gokha, Mabrouk Attia Abd Eldaim

**Affiliations:** 1 Department of Chemistry, Faculty of Science, Menoufia University, Shibin El-Kom 32511, Egypt; sara.fathy.mostafa@gmail.com (S.F.A.E.); chemist_abdullah_2009@yahoo.com (A.A.S.A.); douaaabdelsatar94@gmail.com (D.A.E.); salmaelkady16@yahoo.com (S.E.); amirajulita765@gmail.com (A.E.); tamtamalnakeeb@gmail.com (F.A.); aelgokha@yahoo.com (A.A.E.-G.); 2 Department of Pharmaceutical Chemistry, Faculty of Pharmacy, Helwan University, Cairo 11795, Egypt; shaymaa.taha@pharm.helwan.edu.eg; 3 Research Department, Health Sciences Research Center, Princess Nourah bint Abdulrahman University, Riyadh 84428, Saudi Arabia; 4 Department of Cancer Biology, National Cancer Institute, Cairo University, Cairo 11562, Egypt; saadelgendy2004@yahoo.com; 5 Department of Biochemistry and Chemistry of Nutrition, Faculty of Veterinary Medicine, Menoufia University, Shibin El-Kom 32511, Egypt; mabroukattia@vet.menofia.edu.eg

**Keywords:** BAPPN, HepG2, HCT-116, MCF-7, A549, caspase-3, P53, PCNA, Ki67, VEGF

## Abstract

The current study evaluated the cytotoxic activity of 11-(1,4-bisaminopropylpiperazinyl)5-methyl-5H-indolo[2,3-b]quinoline (BAPPN), a novel derivative of 5-methyl-5H-indolo[2,3-b]quinoline, against hepatocellular carcinoma (HepG2), colon carcinoma (HCT-116), breast (MCF-7), and lung (A549) cancer cell lines and the possible molecular mechanism through which it exerts its cytotoxic activity. BAPPN was synthesized and characterized with FT-IR and NMR spectroscopy. The binding affinity scores of BAPPN for caspase-3 PDB: 7JL7 was −7.836, with an RMSD of 1.483° A. In silico screening of ADME properties indicated that BAPPN showed promising oral bioavailability records in addition to their high gastrointestinal absorption and blood–brain barrier penetrability. BAPPN induced cytotoxicity, with IC_50_ values of 3.3, 23, 3.1, and 9.96 μg/mL against cancer cells HepG2, HCT-116, MCF-7, and A549, respectively. In addition, it induced cell injury and morphological changes in ultracellular structure, including cellular delayed activity, vanishing of membrane blebbing, microvilli, cytoplasmic condensation, and shrunken nucleus with more condensed chromatin autophagosomes. Furthermore, BAPPN significantly increased the protein expression of caspase-3 and tumor suppressor protein (P53). However, it significantly reduced the secretion of vascular endothelial growth factor (VEGF) protein into the medium and decreased the protein expression of proliferation cellular nuclear antigen (PCNA) and Ki67 in HepG2, HCT-116, MCF-7, and A549 cells. This study indicates that BAPPN has cytotoxic action against liver, colon, breast, and lung cancer cell lines via the up-regulation of apoptotic proteins, caspase-3 and P53, and the downregulation of proliferative proteins, VEGF, PCNA, and Ki67.

## 1. Introduction

Cancer occurs because of uncontrolled cell division and growth resulting from tumor suppressor gene inactivation and/or oncogene activation [1]. Roughly, 10 million fatalities, or nearly one in every six deaths, were caused by cancer in 2020, making it the world’s top cause of death [2].

Liver cancer is one of the cancer forms that spreads vastly and more fatally. Liver cancer can be primary or secondary. Primary liver cancer occurs in the liver itself, while secondary liver cancer is a result of the spread of cancer from other organs of the body to the liver [3]. The most frequent type of liver cancer is hepatocellular carcinoma (HCC), which accounts for about 2% of the latest projected cancer cases and 5% of new cancer deaths [4].

The third most frequent cancer and the fourth most prevalent reason for cancer death is colorectal cancer (CRC) [5]. Most CRC cases are found in Western nations, and the prevalence of this disease is rising, as approximately 4–5% of people are diagnosed with colorectal cancer. The main risk factors that increase its incidence are age, history of chronic diseases, and lifestyle choices [5]. Tumor suppressor genes involved in DNA repair pathways and oncogenes are all mutated in CRC [6].

In 2020, 2.3 million new cases of female breast cancer, or nearly 12% of all new cancer cases were reported, making it more prevalent than lung cancer. Additionally, it came in fifth place as the main reason for cancer fatalities. One in four female cancer patients may develop breast cancer, and one in six will die from it. [7]. The risk stratification of women is considered more effective with fewer obstacles that could be considered during the design of breast cancer screening programs [2,8].

Likewise, lung cancer was another primary cause of death in 2021. There were 1.8 million deaths (18.4% of global cancer deaths). Also, it is considered one of the most prevalent types of cancer worldwide (11.6% of overall diagnosed cancer cases), in both men and women. Lung cancer can be categorized into two types: small-cell carcinoma (13% of all lung cancer patients) and non-small-cell carcinoma (83% of all cancer patients) [2,9].

Multiple treatment techniques, such as surgery, systemic anticancer therapy, and radiotherapy, may be used alone or in combination to treat malignancies [10]. The growing knowledge of the biochemical pathways involved in a disease’s processes increases the possibility of developing new approaches to treat cancer. Cancer therapies that are applied include immunotherapy [11], stem cell therapy [12], CAR T-cell therapy [13], gene therapy [14], molecular targeted therapy [15], nano-therapy [16], liposomal drugs [17], hormone therapy [18], and oncotherapy, targeting specific genetic mutations [19]. Although chemotherapy is a successful medication for different types of cancer, it is also associated with several serious side effects, including early indicators of toxicity and late signs of chronic toxicity [20]. All human organs, especially vital ones like the heart, lungs, and brain, are susceptible to damage. Additionally, the long-term effects of chemotherapy include infertility, cancer, and drug resistance [20].

5-methyl-5H-indolo[2,3-b]quinoline is a tetracyclic nitrogen heterocycle that was discovered in the African climber *Cryptolepis sanguinolenta*. It is commonly utilized in Central and West Africa as a traditional medicine [21]. 5-methyl-5H-indolo[2,3-b]quinoline belongs to the small family of indolo[2,3-b]quinoline alkaloids and is one of its characteristic examples. It has a variety of biological properties, including the ability to bind to DNA and acts as topoisomerase II inhibitor [21]. Additionally, it exhibits antiprotozoal, molluscicidal, cytotoxic, antifungal, and antibacterial activities. The precise mechanisms underlying all the observed bioactivities have not been fully elucidated. However, the affinity of the substance to bind to DNA and to impede topoisomerase II has been connected to some of these actions. The studies concerning the 5-methyl-5H-indolo[2,3-b]quinoline activities have focused on its cytotoxic and antimalarial capabilities [22]. Furthermore, 5-methyl-5H-indolo[2,3-b]quinoline analogs provide templates with therapeutic promise and have previously been shown to have significant anticancer and DNA intercalation capacities [23]. Therefore, the current study aimed to assess the cytotoxic effect of 11-(1,4-Bisaminopropylpiperazinyl 5-methyl-5H-indolo[2,3-b]quinoline on liver, colon, breast, and lung cancerous cell lines as well as the potential mechanism by which it exerts its cytotoxic activity and its effect on the ultrastructure of the cancer cell.

## 2. Results

### 2.1. Synthesis of 11-(1,4-Bisaminopropylpiperazinyl)5-methyl-5H-indolo[2,3-b]quinoline **4** (BAPPN) and Its Structural Characterization by ^1^H NMR and ^13^C NMR

**Scheme 1:**
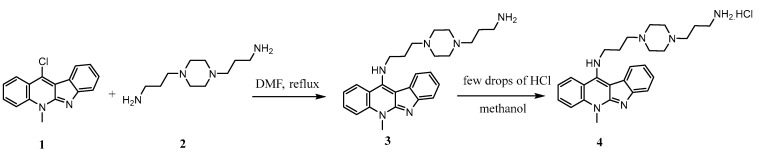
Synthesis and characterization of 11-(1,4-Bisaminopropylpiperazinyl)neocryp-tolepine hydrochloride **4** (BAPPN).

The key starting 11-chloroneocryptoleine **1** reacted with an excessive amount (3 times) of 1.4-bis (3-aminopropyl) piprerazine **2** via nucleophilic substitution rection to obtain free amine **3**. The solution of **3** in methanol was acidified with few drops of 1M of HCl until the formation of the target hydrochloride salt of **4** (BAPPN) as illustrated in Scheme 1.

The synthesized hydrochloride salt of 11-(1,4-bisaminopropylpiperazinyl)5-methyl-5H-indolo[2,3-b]quinoline **4** was elucidated by FTIR and NMR spectroscopic methods. The FTIR spectrum showed the corresponding absorption peak of (NH) at υ 3400 cm^−1^ and (NH2) at υ 3334 cm^−1^ whereas aromatic (CH) appeared at υ 3057 cm^−1^ and aliphatic (CH) at υ 2848 cm^−1^. In addition, υ for (C=CAr) was recorded at 1611 cm^−1^. ^1^H NMR spectrum showed two chemically different multiplet at δ: 2.20 and 2.35 ppm which revealed the presence of methylene protons (-CH_2_-CH_2_-CH_2_-) adjacent to the two carbon atoms in the aliphatic carbon spacer for the two-residual propyl chains in **4**. Furthermore, the methylene protons neighboring to the amino group appeared at δ: 3.09 ppm, whereas the four (CH_2_) protons of piperazine ring showed multiplet at δ: 3.39 ppm. In addition, the singlet at δ: 3.74 ppm was assigned to the acyclic (*N*-CH_2_) protons. On the other hand, the singlet appeared at δ: 4.21 ppm was assigned to the (-CH_2_-NH) directly attached to indoloquinoline core, while the methyl proton of quinoline (*N*-CH_3_) appeared at δ: 4.33 ppm. Finally, the two broad singlet peaks at δ: 12.72 and 12.87 ppm were assigned to (NH_2_) and (NH), respectively. Moreover, the ^13^C NMR spectrum recorded the appearance of eight signals representing the aliphatic carbons. While the other carbon signals were assigned for the aromatic indoloquinoline core structure of **4**.

### 2.2. Modeling Studies

**Figure 1:**
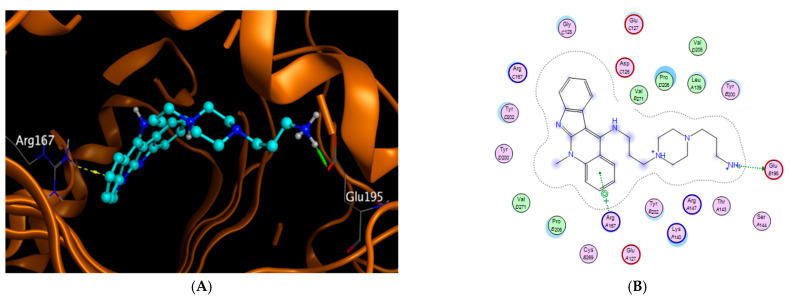
Amino acids involved in interactions between BAPPN in cyan ball and sticks with caspase-3 protein pdb ID 7jl7 in both. (**A**) 3D and (**B**) 2D interactions.

The binding affinity scores of 11-(1,4-bisaminopropylpiperazinyl)5-methyl-5H-indolo[2,3-b]quinoline for caspase-3 PDB: 7JL7 were −7.836 with an RMSD of 1.483° A. The docked 11-(1,4-Bisaminopropylpiperazinyl)5-methyl-5H-indolo[2,3-b]quinoline was totally inside the binding interaction surface; the amino acids involved in interaction were Glu 195 and Arg 167 (Figure 1A,B). The docking results for 11-(1,4-Bisaminopropylpiperazinyl)5-methyl-5H-indolo[2,3-b]quinoline in the downloaded protein along with the chemical bonding in addition to the amino acids involved in the interaction are tabulated in Table 1.

### 2.3. Bioavailability and ADME in Silico Studies

**Figure 2:**
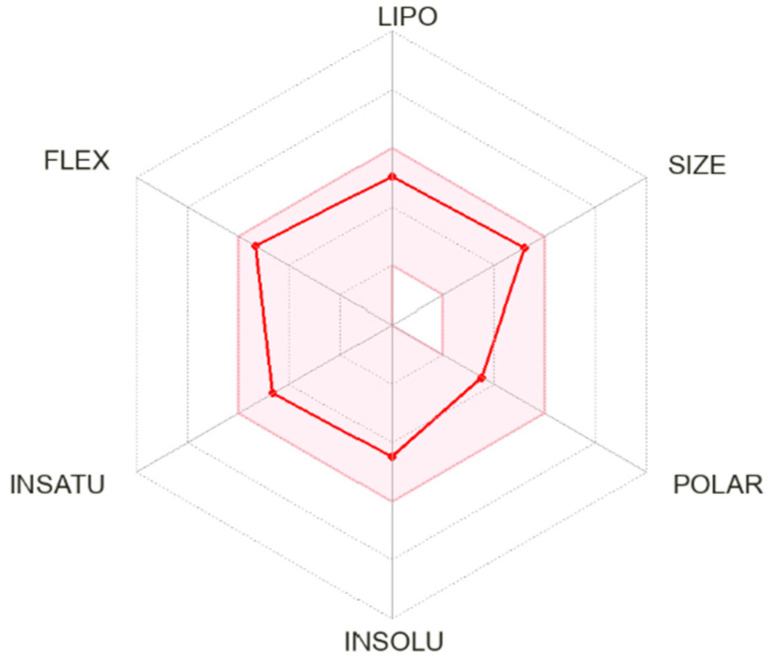
Oral bioavailability for BAPPN was illustrated in the red colored zone.

The pharmacokinetic properties of 11-(1,4-Bisaminopropylpiperazinyl)5-methyl-5H-indolo[2,3-b]quinoline were studied in silico using Swiss ADME http://www.swissadme.ch. Accessed on 21 October 2022. BAPPN has a molecular weight of 430.5 and eight rotatable bonds. Regarding H-bonding, BAPPN has two donors (HBD) and four acceptors (HBA). Its ilog *p* value (3.67) < 5, indicating good toleration to cell membranes (Table 2). In addition, its oral bioavailability was illustrated in (Figure 2), where the best oral bioavailability is within the red zone. BAPPN shows promising oral bioavailability records in addition to their its gastrointestinal absorption and blood–brain barrier permeability records as well.

### 2.4. Drug Likeness Probability

**Figure 3:**
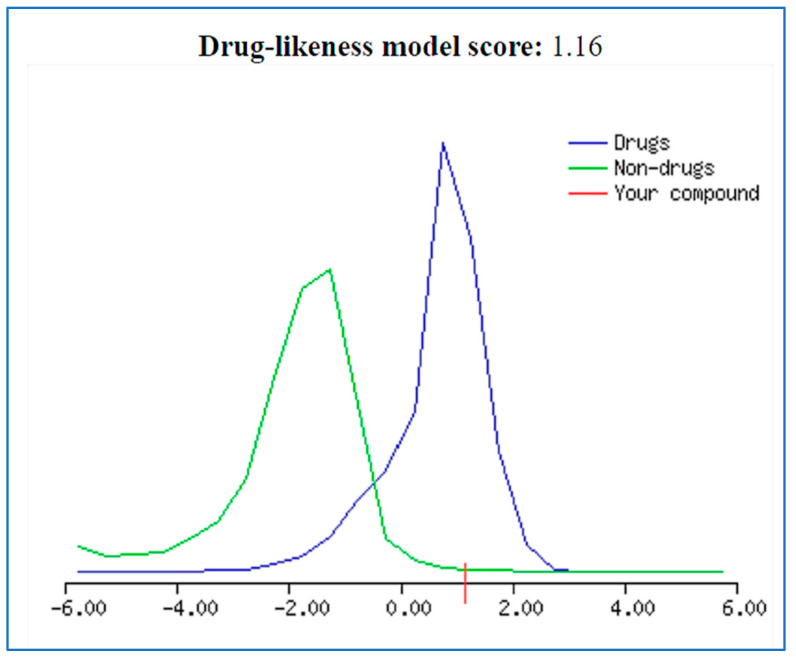
Drug likeness score for BAPPN represented as red stick.

The prepared compound was screened using Molsoft software, Molsoft (www.molsoft.com accessed on 21 October 2022) which declared a very promising drug-like value of 1.16 in the drug-likeness model score (Figure 3).

### 2.5. 11-(1,4-Bisaminopropylpiperazinyl)5-methyl-5H-indolo[2,3-b]quinoline Induced Cytotoxicity in Cancer Cell Lines

**Figure 4:**
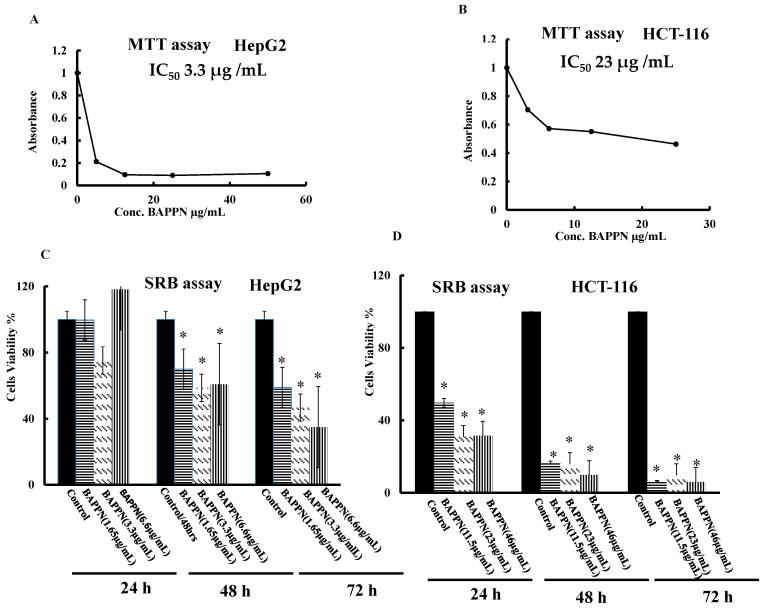
The inhibition concentration (IC_50_) and Cytotoxic Activity of 11-(1,4-Bisaminopropylpiperazinyl)5-methyl-5H-indolo[2,3-b]quinoline (BAPPN). (**A**,**B**) Showed inhibition concentration IC_50_ of BAPPN on HepG2 and HCT-116 cell lines, respectively, cells were treated with different concentrations of BAPPN for 48 h. (**C**,**D**) Showed the viability percentage of HepG2 and HCT-116 cells, respectively when cells treated with BAPPN at different doses of 1.65 μg/mL( half IC_50_), 3.3 μg/mL (equal IC_50_) and 6.6 μg/mL (double IC_50_) for HepG2 cells and 11.5 μg/mL( half IC_50_), 2 3 μg/mL (equal IC_50_) and 46 μg/mL (double IC_50_) for HCT-116 cells. Columns carrying asterisks are significantly at *p* < 0.05 compared to control cells.

Upon treatment of HepG2 cancer cells with BAPPN at gradual doses of 50, 25, 12.5, 5, or 0.0 µg/mL for 48 h and HCT-116 cancer cells at gradual doses of 25, 12.5, 6.25, 3.1, or 0.0 µg/mL for 48 h cytotoxicity was induced with IC_50_ 3.3 µg/mL for HepG2 cells (Figure 4A) and IC_50_ 23 µg/mL for HCT-116 cells (Figure 4B). To confirm the cytotoxicity and determine the effective time and dose of BAPPN, HepG2 and HCT-116 cell lines were treated with BAPPN at doses of 0, 1.65, 3.3, or 6.6 µg/mL medium for HepG2 (Figure 4C) and doses of 0, 11.5, 23, or 46 µg/mL for HCT-116 cells (Figure 4D) for 24, 48 or 72 h. Upon investigating the results, it was found that treatment of HepG2 and HCT-116 cancerous cell lines with BAPPN caused a significant decrease in the percentage of cell viability when compared to the control cells treated with DMSO.

**Figure 5:**
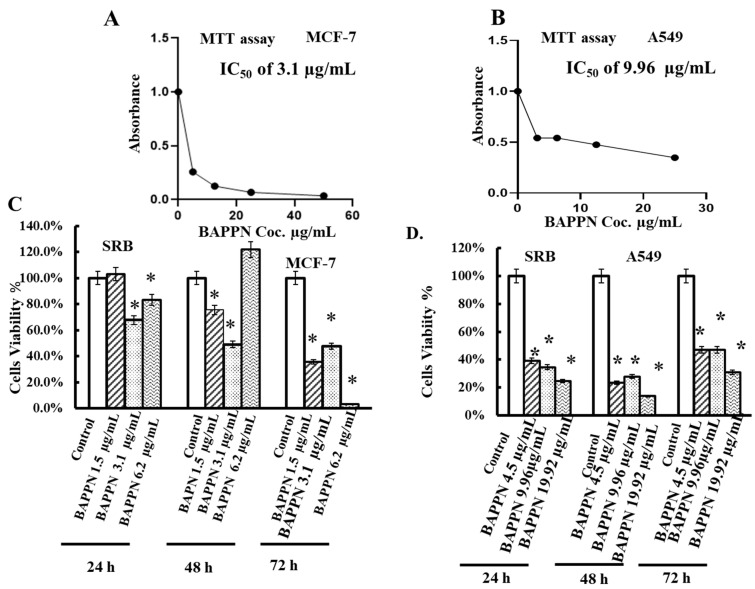
(**A**,**B**) showed the effect of 11(1,4-Bisaminopropylpiperazinyl)5-methyl-5H-indolo[2,3-b]quinoline on MCF-7 and A549 cells lines treated with different concentration for both cells were (1.5, 3.1, 6.2 μg/mL) for MCF-7 and (4.5, 9.96, 19.92 μg/mL) for A549 cells for 48 h. Viability percent and the inhibition concentration IC_50_ of BAPPN compound for (**A**) MCF-7 cells, (**B**) for A549 cells. (**C**) Anticancer activity of BAPPN compound and viability percent using MTT; MCF-7 cells when treated at a different dose of half IC_50_ (1.5 μg/mL), equal IC_50_(3.1 µg/mL), and double IC_50_ (6.2 µg/mL), (**D**) A549 cells treated at dose of half IC_50_ (4.5 µg/mL), equal IC_50_ (9. 96 µg/mL), and double IC_50_ (19.92 μg/mL). Columns carrying asterisks are significant at *p* < 0.05 compared to control cells. Bars represent mean ± SE of 3 values.

In addition, treatment of MCF-7 and A549 cells with BAPPN at doses of 50, 25, 12, 5, or 0 g/mL for 48 h induced cytotoxicity with IC_50_ values of 3.1 µg/mL and 9.96 μg/mL, respectively (Figure 5A,B). Aiming to confirm the cytotoxic effect of BAPPN on breast and lung cell lines, the MCF-7 cell line was treated with BAPPN at doses 0, 1.5, 3.1, or 6.2 μg/mL, and the A549 cell line at doses of 0, 4.5, 9.96, or 19.92 μg/mL for 24, 48, or 72 h. This indicated a significant decrease in the cell viability of both MCF-7 and A549 cells at several doses of BAPPN and at different treatment intervals compared to control cells treated with DMSO as illustrated in the figure below, (Figure 5C,D).

### 2.6. 11-(1,4-Bisaminopropylpiperazinyl)5-methyl-5H-indolo[2,3-b]quinoline Induced Detrimental Changes in the Ultrastructure of Cancer Cell Lines

**Figure 6:**
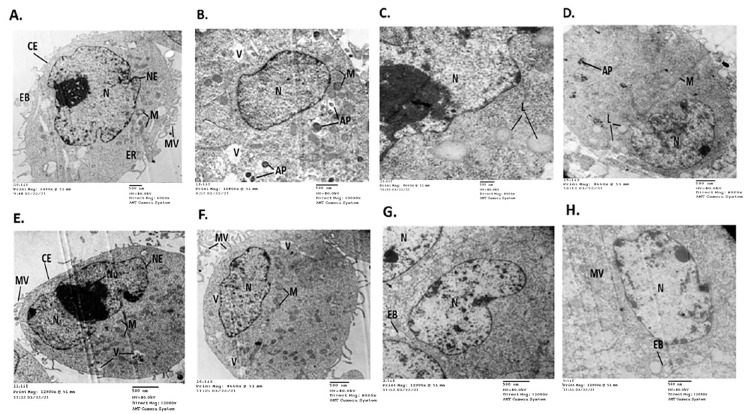
TEM micrograph of ultra-structural characteristic of HepG2 and HCT-116 cells, cells cultured at 96/well microplate and treated with BAPPN at dosage of 3.3 μg/mL for HepG2 cells and dosage of 23 μg/mL for HCT-116 cells for 48 h. (**A**,**B**) Control HepG2 cells (**C**,**D**) treated HepG2 cells. (**E**,**F**) Control HCT-116 cells and (**G**,**H**) treated HCT-116 cells; Scale bar = 500 nm.

TEM images of the control HepG2 cells treated with DMSO revealed normal cancer cells with intact cellular and nuclear membranes, a profusion of microvilli and membrane blebbing on the surface of plasma membranes, and central big nuclei. The cellular organelles including electron-dense pleomorphic mitochondria, lipid droplets (LDs), and endoplasmic reticulum were regularly distributed in the cytoplasm (ER) (Figure 6A,B). Whereas HepG2 cancerous cells treated with BAPPN displayed multiple morphological alterations indicating cell damage and inactivation. These ultrastructural alterations included cytoplasm condensation, reduced microvilli, uneven cell membranes, and membrane blebbing on the cell surface (cm). Oval mitochondria were found to be fragmented, larger, and peripherally aggregated in the cytoplasm. Autophagosomes have been seen (AP), alongside centric tiny nuclei, damaged nuclear membrane, and heterochromatin expression (Figure 6C,D).

Images of the control HCT-116 cells treated with DMSO showed normal distribution of cellular organelles, such as peroxisome-like, electron-dense pleomorphic mitochondria, few cytoplasmic vacuoles, and dense secretory granules. (Figure 6E,F). However, TEM images of the HCT-116 cancer cells treated with BAPPN showed cytoplasmic condensation such that the cytoplasmic and nuclear membranes were disintegrated, mitochondria accumulated, and the apoptotic nucleus had peripherally condensed chromatin. Nucleus fragments and a micronucleus were found (N), microvilli (Mi) vanished, and membrane blebbing was seen (Figure 6G,H).

### 2.7. Effect of 11-(1,4-Bisaminopropylpiperazinyl)5-methyl-5H-indolo[2,3-b]quinoline on the Expression of Apoptotic and Proliferative Proteins in Cancer Cells

To evaluate the possible mechanism through which BAPPN induces cell toxicity, the HepG2 and HCT-116 cell lines were cultured and conditioned with BAPPN at doses of 0 and 3.3 µg/mL as well as 0 and 23 µg/mL respectively, for 48 h. Then, the expression of proapoptotic protein (caspase-3), tumor suppressor protein (P53), angiogenic protein (VEGF), and the proliferation proteins (PCNA and Ki67) were determined by ELISA.

**Figure 7:**
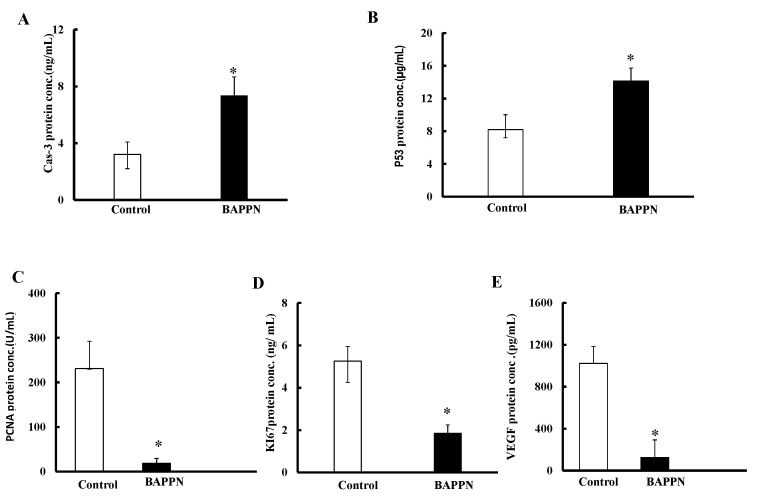
Effect of 11-(1,4-Bisaminopropylpiperazinyl)5-methyl-5H-indolo[2,3-b]quinoline (BAPPN) on the protein expression of caspase-3, P53, PCNA, Ki67, and secretion of VEGF protein in HepG2 cell lines; HepG2 cells were treated with BAPPN at doses of 0 and 3.3µg/mL for 48 h. (**A**) Caspase-3, (**B**) P53, (**C**) PCNA, (**D**) Ki67, (**E**) VEGF. * significant at *p* < 0.05 compared to control cells treated with DMSO.

**Figure 8:**
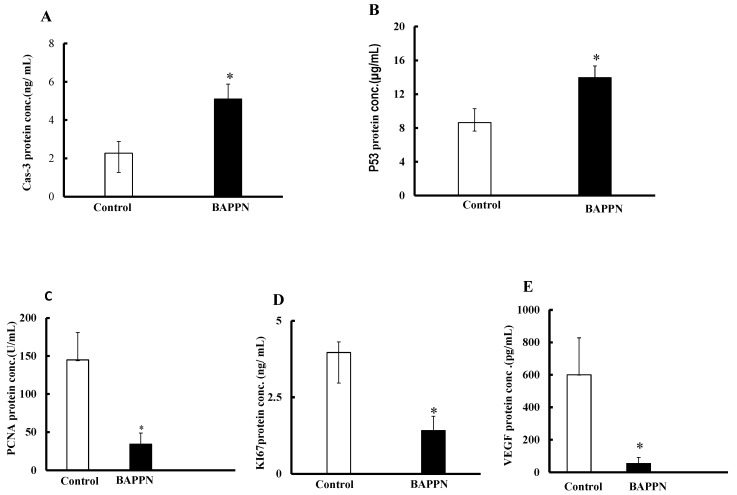
Effect of 11-(1,4-Bisaminopropylpiperazinyl)5-methyl-5H-indolo[2,3-b]quinoline (BAPPN) on the protein expression of caspase-3, P53, PCNA, Ki67, and secretion of VEGF protein in HCT-116 cell lines; HCT-116 cells were treated with BAPPN at doses of 0 and 23µg/mL for 48 h. (**A**) Caspase-3, (**B**) P53, (**C**) PCNA, (**D**) Ki67, (**E**) VEGF. * significant at *p* < 0.05 compared to control cells treated with DMSO.

The treatment of cancer the cell lines with BAPPN significantly increased the protein expression of caspase-3 and P53 in HepG2 cells (Figure 7A,B) and HCT-116 cells (Figure 8A,B) compared to DMSO-treated control cells; however, the treatment downregulated PCNA and Ki67 protein expression in HepG2 cells (Figure 7C,D) and HCT-116 cells (Figure 8C,D) in comparison to DMSO-treated control cells. Moreover, the treatment of cells with BAPPN significantly reduced the secretion of VEGF protein in the medium of HepG2 cells (Figure 7E) and HCT-116 cells (Figure 8E) compared to that of the control cells.

**Figure 9:**
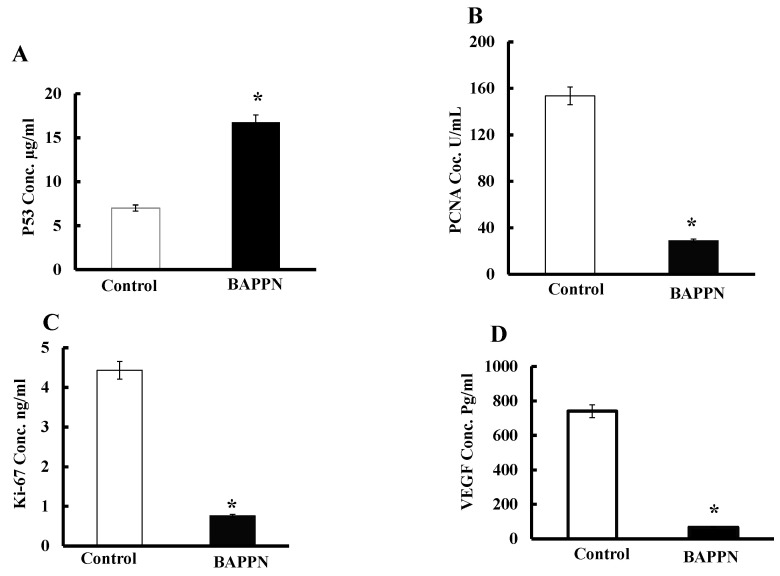
(**A**) showed effect of BAPPN compound on P53 protein level in effect in treated MCF-7cells with BAPPN at 3.1µg/mL for 48 h and control cells, (**B**) PCNA protein level in effect in treated MCF-7 cells with BAPPN at 3.1µg/mL for 48 h and control cells, (**C**) Ki-67 protein level in media for MCF-7 cells treated with 3.1µg/mL BAPPN for 48 h and control cells, (**D**) VEGF protein secretion from MCF-7cells. Columns carrying asterisks are significantly at *p* < 0.05 compared to control cells.

**Figure 10:**
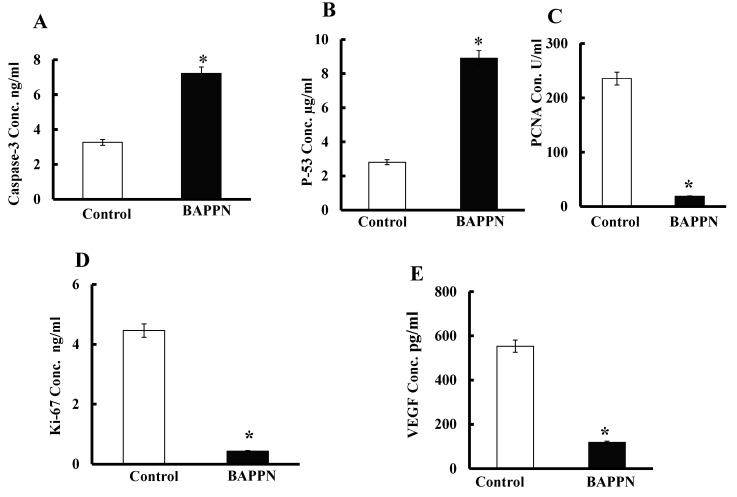
Protein expression in A549 cancer cells treated with 9.96 µg/mL of BAPPN for 48 h compared to DMSO (control cells). (**A**) Caspase-3 protein level, (**B**) P53 protein level, (**C**) PCNA protein level, (**D**) Ki-67 protein level, (**E**) VEGF proteins level. * significant at *p* < 0.05 compared to control cells.

In addition, BAPPN significantly increased the protein expressions of P53 in MCF-7 cells (Figure 9A) and the protein expressions of caspase 3 and P53 in A549 (Figure 10A,B) in comparison with the control treated with DMSO. Additionally, there was a marked decrease in the protein expressions of PCNA and Ki67 in BAPPN-treated MCF-7 cells (Figure 9B,C) and A549 cells (Figure 10C,D). Furthermore, the release of VEGF protein was significantly attenuated in the surrounding medium of MCF-7cells (Figure 9D) and A549 cells (Figure 10E) compared to the control cells.

## 3. Discussion

Due to the high mortality rates linked with liver and colon cancer, a medication that is both non-toxic and safe is needed to treat this terrible illness. Cryptolpine has been shown to cause cell cycle disruption and apoptosis. [24,25]. Additionally, it has been demonstrated that 5-methyl-5H-indolo[2,3-b]quinoline (neocryptolipine) is a promising chemical precursor for the development of novel anticancer drugs with potentially active derivatives [26]; BAPPN is among these derivatives.

The results of the current study revealed that BAPPN had a cytotoxic effect on the HepG2, HCT-116, MCF-7, and A549 cell lines with IC_50_ 3.3 µg/mL for HepG2; 23 µg/mL for HCT-116 (Figure 4A,B); 3.1 µg/mL for MCF-7; and 9.96 µg /mL for the A549 cell lines (Figure 5A,B). These findings matched with the literature, which reported that indoloquinolines (5-methyl-5H-indolo[2,3-b]quinoline derivatives) binds strongly to DNA and that modification of the structure of indoloquinolines by amino alkylation improves their biological activity, selectivity index, and cytotoxic activities [27]. Moreover, in our previous in vitro and in vivo studies of closely similar C-11 substituted amine analogs of indoloquinoline core structure, we had proven that this class of natural based alkaloids had insignificant cytotoxicity on normal cells [23,28].

The cytotoxic activity of BAPPN has been confirmed upon the treatment of the cancer cell lines with different concentrations for different periods, which indicated a reduction in the percentage of cell viability as illustrated in Figure 4C,D and Figure 5. Comparably, it has been reported that the 5-methyl-5H-indolo[2,3-b]quinoline analog had a curative effect on solid tumors with minimal side effect on the liver [23]. Also, a series of 11-substituted cryptolpine derivatives have been previously synthesized and tested for their cytotoxic effect on the HCT-116 colon cancer cell line and Raji lymphoma cells [29]. Rodphon et al. evaluated the cytotoxicity of eighteen isocryptolepine-triazole hybrids, which indicated that some hybrid-isocryptolepine containing triazole linkages resulted in cytotoxicity against the HepG2, HuCCA-1, and A549 cell lines with lower toxicity compared to normal cell line [30].

Moreover, it was reported that the 5-methyl-5H-indolo[2,3-b]quinoline derivative has high selectivity and cytotoxicity against gastric cancer cells compared to the 5-methyl-5H-indolo[2,3-b]quinoline itself [20].

In the current study, the cytotoxic effect of BAPPN was confirmed by examining the ultrastructure and morphology of the HepG2 and HCT-116 cell lines with TEM. Transmission electron micrograph of the control cells of both HepG2 and HCT-116 cells displayed normal cellular process with whole integrity of the cells organelle, in addition to round shaped nucleus and nucleolus chromatin (Figure 6A,B,E,F); meanwhile, HepG2 and HCT-116 cells treated with BAPPN showed feature of early apoptosis, indicated by shrinking and chromatin condensation, while late apoptosis was represented by apoptotic body formation, nuclear collapse, and progressing blebbing membrane (Figure 6C,D,G,H). These results were parallel with that of a previous study which indicated that cryptolpine induced Leishmania donavani cell death, represented by membrane blebbing, swollen mitochondria, marginated chromatin, and fragmented nucleus [24]. Furthermore, the literature indicated that 5-methyl-5H-indolo[2,3-b]quinoline analogs have apoptotic activity against Ehrlich ascites carcinoma (EAC), which is represented by dense basophilic and eosinophilic cytoplasm [23].

The apoptotic effect of BAPPN on the HepG2, HCT-116, MCF-7, and A549 cell lines was induced by increasing the protein expression of casepase3, apoptosis activation factor, and P53, as shown in (Figure 7, Figure 8, Figure 9 and Figure 10). When breast cancer cells are exposed to cytotoxic medicines, radiation, or immunotherapy, caspase-3, a key mediator of death, is activated [31]. Our results were in line with a previous study which demonstrated that Cryptolepis sanguinolenta roots contain two compounds, 5-methyl-5H-indolo[2,3-b]quinoline and cryptolpine, both of which are cytotoxic and act through an apoptotic caspase-3-mediated mechanism [30]. In response to cellular stress, p53 is a crucial mediator of cell cycle arrest [32]. Activated P53 prevents cells with extensive DNA damage from replicating by inducing cell cycle arrest, which allows for DNA repair and/or apoptosis via transactivation of its target genes related to cell cycle arrest and/or apoptosis induction. Therefore, p53’s tumor-suppressing action is closely tied to its DNA-binding activity [33]. Our findings were in line with that of Ozaki T Nakagawara, who demonstrated that inhibiting the production of the mdm2 protein led to p53 activation and accumulation in SCC-13 and A431 cells. [34] Cryptolepine also caused p53 to undergo post-translational changes such as phosphorylation and acetylation.

Furthermore, the other possible cause of the cytotoxic activity of BAPPN is the decrement of the cancer cell lines’ proliferation through downregulation of PCNA and Ki67, which are cell proliferation markers, in HepG2 cells (Figure 7C,D); HCT-116 cells (Figure 8C,D); MCF-7 (Figure 9); and A549 cells (Figure 10). As the proliferating cells spend more time in the G1/S phase, PCNA expression may be utilized as a cell proliferation marker. Additionally, as a factor of DNA repair and replication machinery, this protein is crucial for nucleic acid metabolism [25]. A nuclear protein known as Ki-67 that has been linked to cellular proliferation was first discovered by [35]. Ki-67 nuclear antigen was found to be regulated in cell cycle stages S, G1, G2, and M, but not the G0 phase [36]. These findings agreed with that of [23], who showed that cryptolpine significantly reduced tumor cell proliferation, as evidenced by the significant suppression of PCNA expression. Cryptolpine has been shown to reduce PCNA protein levels. Down-regulation of PCNA and cell cycle arrest are all important components of the apoptotic cascade. In addition, [37] showed that cryptolpine extract was associated with a reduction in Ki67 expression. Moreover, treatment of cells with BAPPN decreased the secretion of VEGF protein into the medium of HepG2 (Figure 7E), HCT-116 (Figure 8E), MCF-7 (Figure 9), and A549 (Figure 10). The signaling pathway for VEGF is crucial in controlling tumor angiogenesis. VEGF is an effective therapeutic target in a variety of human malignancies. Aspriţoiu et al. showed that elevated VEGF expression has been shown to contribute to solid tumor formation by promoting tumor angiogenesis, which is characterized by abnormal angiogenesis, with elevated levels of VEGF production in tumor cells [38]. These findings matched that of [39], who indicated that the IQDMA compound, an indoloquinoline derivative, is an effective antitumor agent against human leukemia cells through the reduction in the expression of VEGF and, consequently, induction of apoptosis in these cells. Furthermore, [31] showed that cryptolpine and 5-methyl-5H-indolo[2,3-b]quinoline were found to have anticancer activity by decreasing VEGF secretion. Therefore, the 5-methyl-5H-indolo[2,3-b]quinoline derivative BAPPN used in the current study is considered a cytotoxic agent against the HepG2, HCT-116, MCF-7, and A549 cell lines via its apoptotic and antiproliferative potential, which matched with the findings of [40] who found that 5-methyl-5H-indolo[2,3-b]quinoline has a unique ability to treat solid tumors.

## 4. Material and Methods

### 4.1. General

The installation of the synthesized hydrochloride salt **4** (BAPPN) was confirmed using spectroscopic techniques such as FT IR and NMR. FT IR spectroscopy in KBr with Alpha-Bruker ATR mode (San Jose, CA, USA) as well as NMR analysis with (400 MHz and 100 MHz) Bruker magnet system 400,54 Ascend/R (Huston, TX, USA) 400 M for ^1^H NMR and ^13^C NMR, respectively, were performed at the Applied Nucleic Acid Research Center, Faculty of Science, Zagazig University. 11-(1,4-Bisaminopropylpiperazinyl)5-methyl-5H-indolo[2,3-b]quinoline (BAPPN) was synthesized in a pure powder form at the Faculty of Science, Menoufia University. It is noteworthy that the key intermediate 11-chloro5-methyl-5H-indolo[2,3-b]quinoline **1** and free amine **3** were prepared as cited in the reported method [40,41]. Without further purification, the solvents were applied as received. Commercial starting materials were obtained from Sigma Aldrich. 3-(4,5dimethylthiazol-2-yl)-2,5-diphenyltetrazolium bromide (MTT), dimethyl sulfoxide (DMSO), absolute methanol, and phosphate-buffered saline (PBS) pH 7.2 were supplied by Sigma-Aldrich (Darmstadt, Germany). Cell culture medium RPMI-1640 containing L-glutamine and fetal bovine serum (FBS) were bought from Gibco^®^ (Thermo Fisher Scientific, Loughborough, UK), while penicillin, streptomycin, and trypsin/EDTA (0.25%) were supplied by Nuaillé, France. ELISA Kits for vascular endothelial growth factor (VEGF), proliferating cell nuclear Antigen (PCAN), cancer suppressor protein (P53), proliferation protein (Ki-67), caspase-3 protein, and NP-40 lysis buffer were purchased from Biospes Company, Chongqing, China. The liver cancer cell line (HepG2), colon (HCT-116), breast (MCF-7), and lung (A549) cancer cell lines were supplied by the National Cancer Institute (NCI, Cairo University, Egypt).

### 4.2. Preparation of N-(3-(4-(3-Aminopropyl)piperazin-1-Yl)propyl)-5-methyl-5H-indolo[2,3-b]quinolin-11-amine hydrochloride) (BAPPN)

Step 1: (2.33 mL, 11.26 mmol) of 1.4-bis (3-aminopropyl) piprerazine **2** in DMF (2 mL) was added to (1 g, 3.75 mmol) of 11-chloro 5-methyl-5H-indolo[2,3-b]quinoline **1** in the presence of (1.30 mL, 11.26 mmol) of triethyl amine under reflux until the consumption of the reactant was achieved, this was monitored using TLC after 2 h. The free amine **3** was collected after reaction mixture filtration, then washed with water after reaching room temperature.

Step 2: For the acidification of **3** in methanol solution, 1 M of HCl was added dropwise until the formation of the hydrochloride salt of **4**. The precipitated salt was filtered and dried to give **4** in pure form as a yellowish-brown powder.

Yellowish brown solid, yield: (0.64 g, 74%), FT IR (KBr) cm^−1^ υ: 3334 (NH), 3173 (NH_2_), 3057 (=CH), 2848 (CH), 1611 (C=C_Ar_), 1578 (C=N). ^1^H NMR (CD_3_OD 400 MHz) δ: 2.20 (br.m, 2H, CH_2_), 2.35 (br.m, 2H, CH_2_), 3.09 (m, 2H, CH_2_), 3.37 (m, 8H, 4CH_2_), 3.74 (br.m, 4H, 2CH_2_), 4.21 (br.m, 2H, CH_2_), 4.33 (s, 3H, *N*-CH_3_), 7.40–8.65 (m, 8H, CH_Ar_), 12.72 (br.s, 2H, NH_2_), 12.87 (br.s, 1H, NH). ^13^C NMR (CD_3_OD, 100 MHz) δ: 20.34, 23.28, 26.24, 35.65, 36.05, 37.91, 46.63, 55.15, 113.20, 114.63, 118.73, 121.78, 122.11, 124.10, 124.30, 124.50, 125.46, 126.24, 127.81, 129.18, 134.30, 135.02, 138.39, 139.10, 139.46, 149.66, 150.78. (c.f. Supplementary Materials).

### 4.3. Modeling Studies

Molecular docking studies were demonstrated on the 11-(1,4-Bisaminopropylpiperazinyl)5-methyl-5H-indolo[2,3-b]quinoline to investigate their affinity for caspase-3 enzymes. A molecular operating environment (MOE.2014.09) was used to perform molecular docking according to [42], and caspase-3 protein was downloaded from the protein data bank (pdb: 7JL7) [43]. Ligand and protein structural optimizations were applied as reported [44,45] by calculating partial charges, 3D protonation, and strands correction followed by energy minimization.

The selected docking protocol was induced fit; the active site was selected at dummy atoms where we applied alpha spheres and backbones as a placement guide. We browsed the database of the investigated compound in mdb format, and pharmacophore annotations were excluded. The gradient for energy was minimized to 0.05, and the force field was chosen as MMFF94X by the system default [46].

### 4.4. Pharmacokinetics in Silico-Screening

11-(1,4-Bisaminopropylpiperazinyl)5-methyl-5H-indolo[2,3-b]quinoline was studied for its pharmacokinetic properties and oral bioavailability in silico using Swiss ADME [47,48], while drug likeness was investigated using Molsoft online.

### 4.5. Cell Culture

The HepG2, HCT-116, MCF-7, and A549 cell lines were cultured in cell culture plates at a density of 1.5 × 10^5^/well in RPMI-1640 medium with L-glutamine, FBS 10%, and penicillin/streptomycin 1%, which was maintained at 37 °C and 5% CO_2_ for 48 h until the cell confluence reached 75%. Then, cells were harvested by using trypsin EDTA 0.25% and then reseeded again to make cell stocks.

### 4.6. Determination of Medium Inhibitory Concentration (IC_50_) by MTT Assay

The HepG2, HCT-116, MCF-7, and A549 cell lines were cultured in 96-well culture plates at a density of 1.5 × 10^4^/well in RPMI-1640 medium containing L-glutamine, FBS 10%, and penicillin/ streptomycin 1%, and incubated at 37 °C and 5% CO_2_ for 24 h. Then, the culture medium was aspirated and replaced with RPMI-1640 medium containing 10% FBS, 1% penicillin/streptomycin, and 50, 25, 12.5, 5, or 0 µg/mL BAPPN to treat HepG2, MCF-7, and A549 25, 12.5, 6.25, 3.1, and 0 µg/mL of BAPPN for the HCT-116 cell lines for 48 h at 5% CO_2_ and 37 °C. The median inhibitory concentrations (IC_50_) for the HepG2, HCT-116, MCF-7, and A549 cell lines were determined by GraphPad prism version 6 according to [49].

### 4.7. Sulforhodamine B Assay (SRB Assay)

To confirm the cytotoxicity of BAPPN on the HepG2 and HCT-116 cell lines, cells were seeded in 96-well microtiter culture plates at a density of 10^4^/well in RPMI-1640 medium containing L-glutamine, FBS (10%), and penicillin/streptomycin 1%. This was maintained for 24 h in a humidified 5% CO_2_ incubator at 37 °C. Then, the medium was changed and the HepG2 cell line was treated with BAPPN at doses of 0 µg/mL, 1.65 µg/mL (half of IC_50_), 3.3 µg/mL (equal IC_50_), and 6.6 µg/mL (double IC_50_) for 24, 48, and 72 h, respectively. HCT-116 cells were conditioned with BAPPN at different concentrations 0 µg/mL, 11.5 µg/mL (half of IC_50_), 23 µg/mL (equal IC_50_), and 46 µg/mL (double IC_50_) for 24, 48, and 72 h. MCF-7 cells were treated with BAPPN at doses of 0, 1.5 μg/mL (½ IC_50_), 3.1 (IC_50_), and 6.2 μg/mL (double IC_50_) for 24, 48, and 72 h. A549 cells were treated with BAPPN at doses 0, 4.5 (½IC_50_), 9.96 (IC_50_), and 19.92 μg/mL (double IC_50_) and incubated for 24, 48, and 72 h. Then, the treated cells were fixed with 10% trichloroacetic acid (TCA) for 1 h at 4 °C, and then exposed to 0.4% SRB solution for 10 min in the dark. They were then rinsed with 1% glacial acetic acid after several washings with phosphate-buffered saline (PBS). The formed SRB-stained cells were dissolved by Tris-Hcl after being dried overnight, and a microplate reader was used to measure the color intensity at 540 nm wavelength [50]. Based on the following calculation, the viability percentage was calculated: (Absorbance of sample/Absorbance of control) × 100 = Viability percentage. All experiments were conducted three times in triplicate.

### 4.8. Measurement of the Expression of Cellular Proteins of PCNA, Ki67, P53, VEGF, and Caspase-3 by ELISA Assay

The HepG2, HCT-116, MCF-,7 and A549 cell lines were seeded in 96-well plates at 104/well density with RPMI-1640 medium with L-glutamine, FBS (10%), and penicillin/streptomycin (1%), and incubated in a humidified 5% CO_2_ incubator at 37 °C for 24 h. Then, the medium was changed and the HepG2 cells were treated with BAPPN at doses of 0 and 3.3 µg/mL; HCT-116 cells were treated with BAPPN at doses of 0 and 23 µg/mL for 48 h; MCF-7 cells were treated with BAPPN at doses of 0 and 3.1 µg/mL for 48 h; and A549 cells were treated with BAPPN at 0 and 9.96 µg/mL for 72 h. The protein concentrations of proliferating cellular nuclear Antigen (PCNA) [51], Ki67 [52], tumor suppressor protein (P53) [53], and caspase-3 [54] in the treated cells, as well as the concentration of vascular endothelial growth factor (VEGF) [55] in the media, were measured using ELISA kits according to manufacturer’s instructions. The PCNA, Ki67, P53, caspase-3, and VEGF polyclonal antibodies were pre-coated onto the 96-well plates of these ELISA kits provided by Biovision, Milpitas, CA, USA, according to the manufacturer’s instructions. The anti-PCNA, Ki67, P53, caspase-3, and VEGF polyclonal antibodies were the primary antibodies and they were biotin conjugated. Following the addition of the standards, test samples, and biotin-conjugated detection antibody, wash buffer was used to wash the wells. After adding the Avidin–Biotin-Peroxidase complex, a wash buffer was used to remove any unbound conjugates. To observe the HRP enzymatic process, TMB substrates were utilized. TMB was catalyzed by HRP to create a blue color product; when the acidic stop solution was added it became yellow. The density of yellow color was relational to the protein concentration. The absorbance was measured at 450 nm wavelength by a microplate reader (Biospes, Chongqing, China). A standard curve was established for each assay and then the concentrations of the above-mentioned proteins were calculated by equations obtained from the standard curves.

### 4.9. Transmission Electron Microscope

For the examination of the ultrastructure of the HepG2 and HCT-116 cell lines, transmission electron microscopy (TEM) was used. The HepG2 and HCT-116 cell lines were cultured in a tissue culture flask with density in RPMI-1640 medium complemented with L-glutamine, FBS (10%), and penicillin/streptomycin (1%). They were incubated in a humidified 5% CO_2_ incubator at 37 °C for 24 h. HepG2 was treated with BAPPN at concentrations of 0 and 3.3 µg/mL, while HCT-116 cells were treated with BAPPN at doses of 0 and 23 µg/mL for 48 h. Then, the cells were passed through a series of ethanol and acetone solutions for 30 min after the suspension had been centrifuged down to a pellet and had been rinsed twice with phosphate-buffered saline (PBS) for two hours. Uranyl acetate and lead citrate were used to stain ultrathin sections. The samples were evaluated by a JEOL–JEM 1010 TEM, Tokyo, Japan-made [56].

### 4.10. Statistical Analysis

The data were presented as mean ±SEM. One-way ANOVA was used for the statistical analyses of the SRB experiment, followed by Duncan’s post hoc analysis (Chicago, IL, USA). The significance of the differences in the flowcytometry and ELISA results were assessed using an unpaired test. The differences were considered statistically significant at *p* < 0.05.

## 5. Conclusions

This study revealed that 11-(1,4-Bisaminopropylpiperazinyl)5-methyl-5H-indolo[2,3-b]quinoline (BAPPN) has cytotoxic potential against liver, colon, breast, and lung cancer cell lines via activation of caspase-3 and p53, and deactivation of PCNA, Ki67, and VGEF protein expression. Moreover, it induced ultrastructure changes in the cancer cells. Further studies in animal models are needed to investigate the possibility of using BAPPN as an anticancer agent to pave the way for further clinical studies.

## Figures and Tables

**Table 1:** Docking results for 11-(1,4-Bisaminopropylpiperazinyl)5-methyl-5H-indolo[2,3-b]quinoline hydrochloride in caspase-3.

Compound	Protein	PDB Code	Binding Energy	RMSD	Amino Acids	Type of Bonding
BAPPN	Caspase-3	7JL7	−7.836	1.483	GLU195	H-donor ionic
					ARG 167	*Pi*-cation

**Table 2:** Bioavailability and ADME values for BAPPN upon Swiss ADME screening.

M.wt	Rotatable Bonds	HBA	HBD	TPSA	IlogP	GI Absorption	BBB Permeant	Lipinski Violations	Bioavailability Score
430.5	8	4	2	62.3	3.67	High	Yes	0	0.55

## Data Availability

All data used in this study are included in this Manuscript.

## Supplementary Materials

The supporting information can be downloaded at: https://www.mdpi.com/article/10.3390/ijms241814336/s1.
