# Supplementary material for: Cytotoxic Potential of Novel Quinoline Derivative: 11-(1,4-Bisaminopropylpiperazinyl)5-methyl-5H-indolo[2,3-b]quinoline against Different Cancer Cell Lines via Activation and Deactivation of the Expression of Some Proteins
Source: Int J Mol Sci. 2023 Sep 20;24(18):14336. doi: 10.3390/ijms241814336 (PMC10532317; doi:10.3390/ijms241814336)
Supplement: Supplementary file 1 [file ijms-24-14336-s001.zip › ijms-2554871-supplementary.pdf]

Supporting information

Cytotoxic Potential of Novel Quinoline Derivative 11-(1,4-Bisaminopropylpiperazinyl)5-Methyl-5H-indolo[2,3-b]quinoline against Different Cancer Cell Lines via Activation and Deactivation of the Expression of Some Proteins

Sara Fathy Abd Elrahman [1], Abdullah A. S. Ahmed [1], Doaa Abd Elsatar [1], Salma Elkady [1], Amira Elgendy [1], Fatma Alnakeeb [1], Elshaymaa I. Elmongy [2], Hanan A. Henidi [3,*], Saad M. El-Gendy [4], Ibrahim El Tantawy El Sayed [1,*], Ahmed A. El-Gokha [1] and Mabrouk Attia Abd Eldaim [5]

[1] Department of Chemistry, Faculty of Science, Menoufia University, Shibin El-Kom 32511, Egypt; sara.fathy.mostafa@gmail.com (S.F.A.E.); chemist_abdullah_2009@yahoo.com (A.A.S.A.); douaaabdelsatar94@gmail.com (D.A.E.); salmaelkady16@yahoo.com (S.E.); amirajulita765@gmail.com (A.E.); tamtamalnakeeb@gmail.com (F.A.); aelgokha@yahoo.com (A.A.E.-G.)

[2] Department of Pharmaceutical Chemistry, Faculty of Pharmacy, Helwan University, Cairo 11795, Egypt; shaymaa.taha@pharm.helwan.edu.eg

[3] Research Department, Health Sciences Research Center, Princess Nourah bint Abdulrahman University, Riyadh 84428, Saudi Arabia

[4] Department of Cancer Biology, National Cancer Institute, Cairo University, Cairo 11562, Egypt; saadelgendy2004@yahoo.com

[5] Department of Biochemistry and Chemistry of Nutrition, Faculty of Veterinary Medicine, Menoufia University, Shibin El-Kom 32511, Egypt; mabroukattia@vet.menofia.edu.eg

* Correspondence: hahenidi@pnu.edu.sa (H.A.H.); ibrahimtantawy@science.menofia.edu.eg (I.E.T.E.S.)

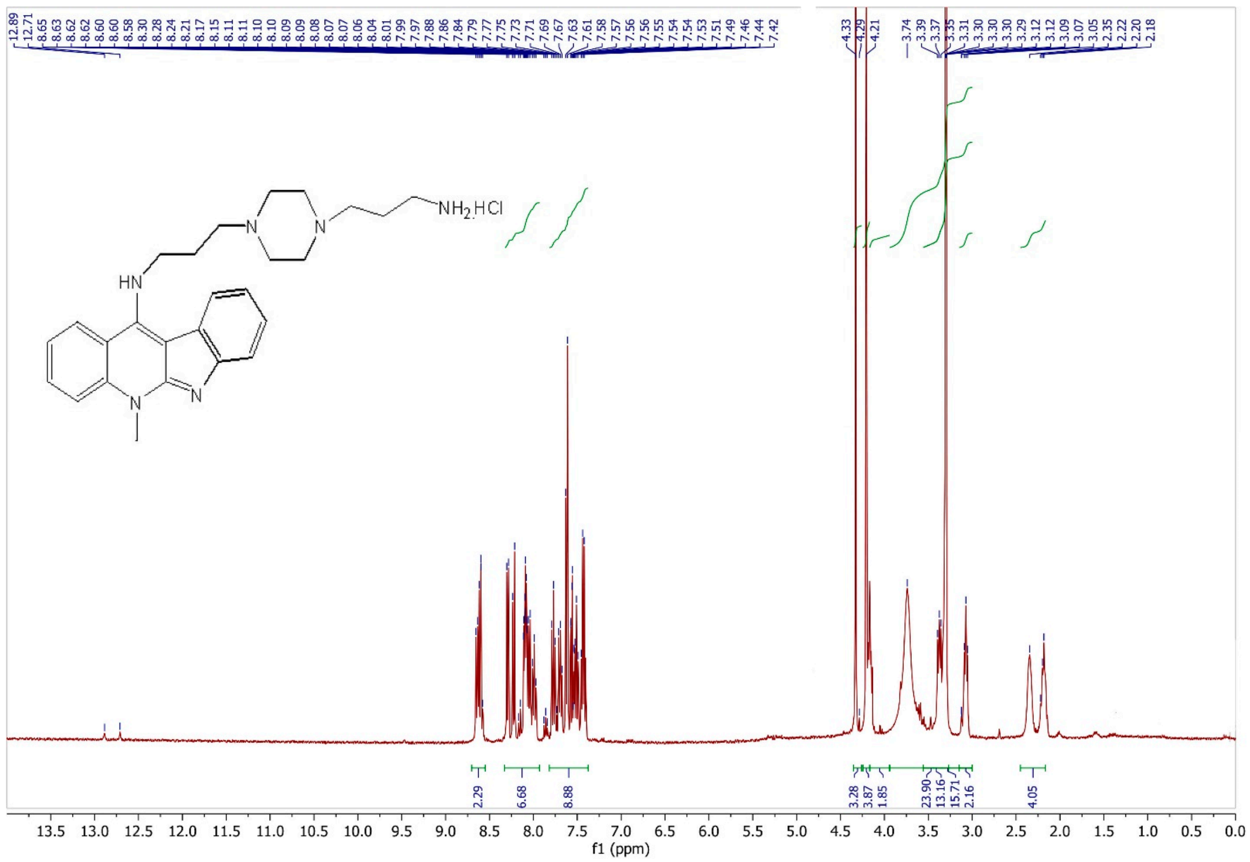

^{1}H NMR of 11(1,4-Bisaminopropylpiperazinyl) indoloquinoline (BAPPN)

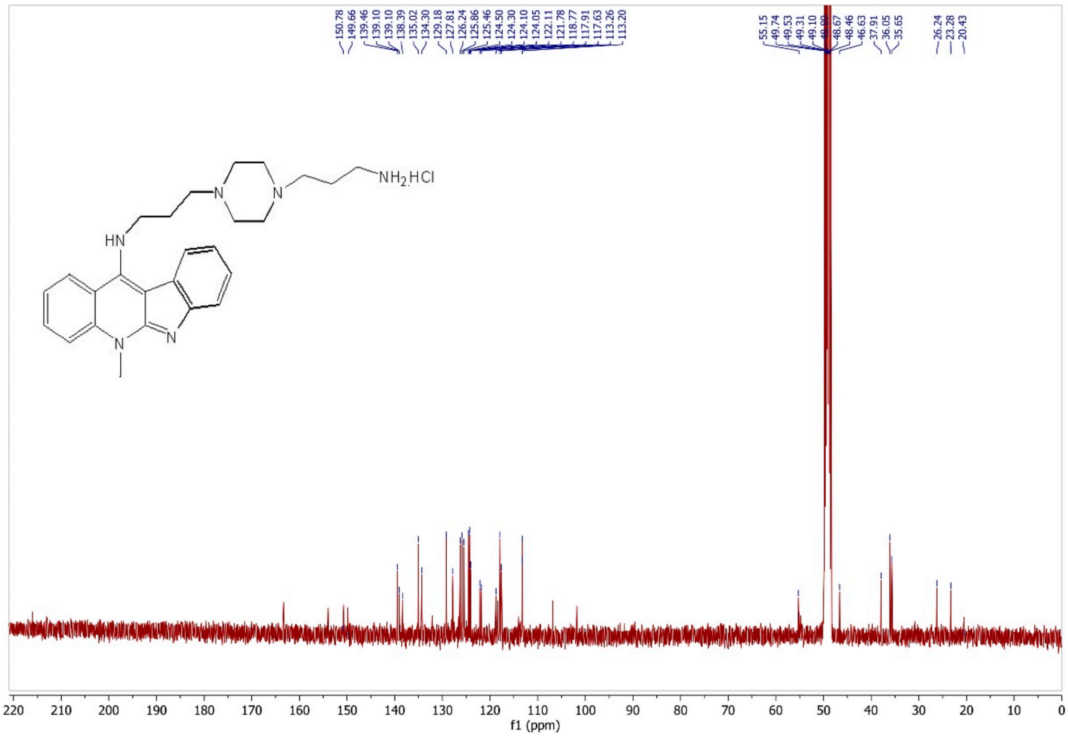

^{13}C NMR of 11(1,4-Bisaminopropylpiperazinyl) indoloquinoline (BAPPN)
